# A Longitudinal Study on the Influences of Self-Esteem and Self-Efficacy in the Teaching Profession on Aspiration to Become Teachers Among University Students in Teaching Programs

**DOI:** 10.1192/j.eurpsy.2026.11491

**Published:** 2026-07-31

**Authors:** K. Yamasaki, K. Uchida

**Affiliations:** Department of Psychology and Educational Science, Narurto University of Education, Naruto, Tokushima, Japan

## Abstract

**Introduction:**

In recent years, an increasing number of teachers in Japan have taken leaves of absence due to mental health problems, largely attributed to the substantial stress of managing students’ psychological and behavioral difficulties, as well as increasingly adverse working conditions. Research suggests that reduced levels of self-esteem and self-efficacy in the teaching profession are linked to this trend, with general self-esteem influencing both domain-specific self-esteem and self-efficacy. As developing these psychological resources can be particularly difficult under such adverse environments, it is crucial to foster self-esteem and self-efficacy in the teaching profession among university students who aspire to become teachers.

**Objectives:**

The present study aimed to examine the influence of general self-esteem, and self-esteem and self-efficacy in the teaching profession on the aspiration to become teachers among university students enrolled in a teacher training program.

**Methods:**

Participants completed the survey twice, once in their first year and again in their second year of university, approximately one year apart. The final sample comprised 193 students (62.2 % female) enrolled in a national teacher-training university in a regional city, all of whom were preparing to become teachers.The measures included the Self-Esteem and Self-Efficacy Scales in the teaching profession, the Intrinsic Self-Esteem Scale for general self-esteem, and an item assessing aspiration to become a teacher. The survey was conducted online.

**Results:**

After confirming high internal consistency for all scales, the hypothesized causal relationships among the variables were examined using path analysis. Regarding self-efficacy in the teaching profession, general self-efficacy and the three subscales (classroom and teaching management, student guidance, and collaboration with others) were examined separately in the model. Because general self-efficacy and the subscales were highly correlated, they could not be entered simultaneously in the path model. The models demonstrated an acceptable fit, with all path coefficients being significantly positive. The results supported a sequential pathway in which general self-esteem predicted self-efficacy in the teaching profession, which in turn predicted self-esteem in the teaching profession, ultimately leading to aspiration to become a teacher.

**Conclusions:**

The level of aspiration to become a teacher was influenced by self-esteem and self-efficacy in a sequential order: general self-esteem, self-efficacy in the teaching profession, and self-esteem in the teaching profession. These findings suggest that fostering self-esteem and self-efficacy related to the teaching profession is essential for university students in teacher training programs to realize their ambition to become teachers.

**Disclosure of Interest:**

None Declared

